# Disentangling associations between DNA methylation and blood lipids: a Mendelian randomization approach

**DOI:** 10.1186/s12919-018-0119-8

**Published:** 2018-09-17

**Authors:** Sergi Sayols-Baixeras, Hemant K. Tiwari, Stella W. Aslibekyan

**Affiliations:** 1 Cardiovascular Epidemiology and Genetics Research Group, IMIM (Hospital del Mar Medical Research Institute), 88 Dr. Aiguader Street, 08003 Barcelona, Catalonia Spain; 2 Universitat Pompeu Fabra (UPF), 80 Dr. Aiguader Street, 08003 Barcelona, Catalonia Spain; 3 CIBER Cardiovascular Diseases (CIVERCV), 88 Dr. Aiguader Street, 08003 Barcelona, Catalonia Spain; 4 Department of Biostatistics, School of Public Health, University of Alabama at Birmingham, 1665 University Blvd, Birmingham, AL USA; 5 Department of Epidemiology, School of Public Health, University of Alabama at Birmingham, 1665 University Blvd, Birmingham, AL USA

## Abstract

**Background:**

DNA methylation is an epigenetic mechanism that has been proposed as a possible link between genetic and environmental determinants of disease. Prior studies reported robust associations between the methylation of specific cytosine-phosphate-guanine (CpG) sites and plasma lipids, namely triglycerides (TGs) and high-density lipoprotein cholesterol (HDL-C). However, the causality of the observed association remains elusive, hampered by weak instrumental variables for methylation status.

**Aim:**

We present a novel application of the elastic net approach to implement a bidirectional Mendelian randomization approach to inferring causal relationships between candidate CpGs and plasma lipids in GAW20 data.

**Methods:**

We used DNA methylation, TGs, and HDL-C measured during the visit 2. Based on prior findings, we selected 5 methylation markers (cg00574958, cg07504977, cg06690548, cg19693031, and cg03717755) related to TGs, 2 markers (cg09572125 and cg02650017) related to HDL-C, and 2 markers (cg06500161 and cg11024682) related to both traits. We implemented an elastic net approach to improve the selection of the genetic instrument for the methylation markers, followed by bidirectional Mendelian randomization 2-stage least-squares regression.

**Results:**

We observed causal effects of blood fasting TGs on the methylation levels of cg00574958 (*CPT1A*) and cg06690548 (*SLC7A11*). For cg00574958, our findings were also consistent with the reverse direction of association, that is, from *CPT1A* methylation to TGs.

**Conclusions:**

Current evidence does not rule out either direction of association between the methylation of the cg00574958 *CPT1A* locus and plasma TGs, highlighting the complexity of lipid homeostasis. We also demonstrated a novel approach to improve instrument selection in DNA methylation studies.

## Background

Fasting blood lipids are independent modifiable risk factors for cardiovascular disease, the leading cause of death worldwide [1, 2]. Like many other complex traits, fasting blood lipids have a heritable component, but known DNA sequence variants only explain a small (< 12% cumulatively) proportion of their variation [3]. An emerging body of evidence supports DNA methylation, which refers to the addition of a methyl group to the DNA molecule, as a more promising contributor to the missing heritability of lipids [4–7]. For example, methylation of one locus in *CPT1A* explained 11.6% of plasma triglyceride variation in a prior epigenome-wide study in the Genetics of the Lipid Lowering Drugs and Diet Network (GOLDN) [4].

In contrast to DNA sequence variants that are inherited from parents and persist through the offspring’s lifetime, methylation markers can be inherited as well as modified by lifestyle and environmental factors [8]. Therefore, the associations reported in previous cross-sectional epigenetic studies of fasting blood lipids have a variety of possible causal interpretations [9]. One method to test the specific causal scenarios (eg, lipids affecting methylation patterns or vice versa) is Mendelian randomization (MR), which uses genetic markers (single-nucleotide polymorphisms [SNPs]) as instrumental variables, taking advantage of the natural randomization that occurs at conception [10]. A study by Dekkers et al. [7] implemented stepwise MR to establish the causal effect of lipids on methylation; however, the presented approach was not truly bidirectional as it was limited in selecting instrumental variables for methylation (ie, *cis*-methylation quantitative trait loci [*cis*-meQTL]). Therefore, the reverse effect of methylation on lipids has not been rigorously tested and cannot be ruled out.

Using data from the GAW20, we aimed to fully interrogate bidirectional relationships between plasma lipids and methylation at 5 methylation markers related to triglycerides (TGs), 2 related to high-density lipoprotein cholesterol (HDL-C), and 2 related to both traits, with selection based on prior evidence [6]. Furthermore, we present a novel approach for selecting SNP proxies for epigenomic variants, using GAW20 data to test the potential of penalized regression, specifically elastic net models, to identify *cis*-meQTL instruments.

## Methods

### Phenotypes and covariates

We used TGs and HDL-C measured during the visit 2 as the phenotypic traits of interest. Both traits were log transformed to normalize their distributions. We selected 5 cytosine-phosphate-guanine (CpG) sites (cg00574958, cg07504977, cg06690548, cg19693031, and cg03717755) related to TGs, 2 CpG sites (cg09572125 and cg02650017) related to HDL-C, and 2 CpG sites (cg06500161 and cg11024682) related to both lipid measures in a previous study from our group [6]. These CpG sites are located in the genes *CPT1A*, *SLC7A11*, *TXNIP*, *MYLIP*, *SYNGAP1*, *PHOSPHO1*, *ABCG1,* and *SREBF1,* and an intergenic region on chromosome 10, respectively. During the analyses, we included age, sex, center, and smoking status as fixed effects, and the family relatedness as a random effect.

### Analysis pipeline

We applied the MR method to interrogate the causal association between lipid traits and DNA methylation. The MR method is predicated upon several assumptions: (1) a reliable association between the genetic instrument and the exposure; (2) associations between the instrument and the outcome must only be mediated through exposure; (3) no pleiotropic effects of the instrument [7, 11]. In the first step of our analysis, we investigated associations between the selected CpG sites and the lipid traits of interest in the GAW20 data. Second, we verified assumption (1) by evaluating associations between a previously validated polygenic risk score as an instrument for lipids (PRS-L) [7] and DNA methylation in the GAW20 data set. Third, we ensured that our polygenic risk score was not associated with methylation other than through its effect on lipid levels, testing assumption (2). To that end, we fitted 2 models, adjusted and unadjusted, for the lipids predicted by the PRS-L. Fourth, we investigated the possibility of reverse causality using a polygenic risk score as an instrumental variable for DNA methylation (PRS-M), which we built using an elastic net approach (detailed below), and testing its effect on lipids. Finally, we assessed the net unmeasured pleiotropic effects [assumption (3)] using the Egger test [12]. For a truly bidirectional approach, we applied these steps in the opposite direction (from methylation to lipids) for all CpG sites that met the Bonferroni threshold (0.05/number of tests) in the first step of the analysis.

### Associations between DNA methylation and lipids

Using the *nlme::R* package [13], we fitted a linear mixed model with DNA methylation beta score as the independent variable and the lipids as the dependent variables, adjusting for the covariates as described above. Methylation status of specific CpG sites was deemed to be significantly associated with lipids if the *p* values met the Bonferroni cutoff of 0.05/7 CpG sites = 0.0071.

### Causal effects of lipids on DNA methylation

We evaluated the causal effects of lipids on DNA methylation using the two-stage least-squares (TSLS) approach [10]. Briefly, TSLS comprises 2 regression stages. In the first stage, the exposure (lipids) is regressed on the genetic instrument (PRS-L) to obtain the values of the exposure predicted by the genetic instrument (lipids|PRS-L). In the second stage, the outcome (DNA methylation) is regressed on the predicted values for the exposure (lipids|PRS-L) from the first stage. Thus, in this second regression, the causal coefficient is estimated [14].

First, we modified a previously validated genetic risk score for lipids [7] based on the availability of its constituent SNPs in GAW20 data. We used 20 available SNPs on the GAW20 data, out of the 28 SNPs proposed by Dekkers et al. [7]. Of these 20 SNPs, 8 were genotyped in GAW20 and 12 were proxy SNPs selected by SNAPtool with an r^2^ > 0.8 [15]. Once we selected the SNPs, we built the PRS-L as $\frac{\sum_{\mathrm{i}=1}^{\mathrm{N}}{genotype}_{\mathrm{i}}\cdotp \mathrm{E}{\mathrm{S}}_{\mathrm{i}}}{\mathrm{mean}\left(\mathrm{ES}\right)}$, where *genotype* is the number of risk alleles carried at a given locus, *N* is the number of SNPs used to build the PRS-L, and *ES* is the effect size. We scaled the PRS-L to obtain a mean of 0 and SE of 1.

Second, we applied the TSLS to estimate the causal effects of lipids on DNA methylation. The first regression was fit to test the association between PRS-L and lipids using a linear mixed-model approach adjusted for the covariates according to the following equation:

$$ {\mathrm{predict}}_{\mathrm{L}}=\overline{\upbeta_0+{\upbeta}_1\ast \mathrm{PRS}\hbox{-} \mathrm{L}+{\upbeta}_2\ast \mathrm{age}+{\upbeta}_{\mathrm{a}}\ast \mathrm{sex}+{\upbeta}_4\ast \mathrm{center}+{\upbeta}_5\ast \mathrm{smoking}}+\overline{\overline{\upbeta_6\ast \mathrm{family}}} $$

(throughout this article, the single line over the text refers to fixed effects and the double line refers to random effects).

The second regression model estimated the causal effect of circulating lipids on DNA methylation:

$$ {\displaystyle \begin{array}{l}\mathrm{CpG}\ \mathrm{site}\ \mathrm{methylation}\\ {}=\overline{\upbeta_0+{\upbeta}_1\ast {\mathrm{predict}}_{\mathrm{L}}+{\upbeta}_2\ast \mathrm{age}+{\upbeta}_3\ast \mathrm{sex}+{\upbeta}_4\ast \mathrm{center}+{\upbeta}_5\ast \mathrm{smoking}}\\ {}+\overline{\overline{\upbeta_6\ast \mathrm{family}}}\end{array}} $$

We also tested whether PRS-L was associated with methylation independently of predicted lipids using the following model:

$$ {\displaystyle \begin{array}{l}\mathrm{CpG}\ \mathrm{site}\ \mathrm{methylation}\\ {}=\overline{\upbeta_0+{\upbeta}_1\ast \mathrm{PRS}\hbox{-} \mathrm{L}+{\upbeta}_2\ast \mathrm{age}+{\upbeta}_3\ast \mathrm{sex}+{\upbeta}_4\ast \mathrm{center}+{\upbeta}_5\ast \mathrm{smoking}+{\upbeta}_6\ast {\mathrm{predict}}_{\mathrm{L}}}\\ {}+\overline{\overline{\upbeta_7\ast \mathrm{family}}}\end{array}} $$

### Causal effects of DNA methylation on lipids

To determine the causal effect of methylation on lipids, we followed the same TSLS approach, starting with selecting the appropriate instrument for methylation. We selected all the SNPs located ±50 kb from the methylation marker as possible *cis*-meQTL. Then we fitted the linear mixed models to obtain the residuals of the association between methylation and the covariates as follows:

$$ {\displaystyle \begin{array}{l}\mathrm{CpG}\ \mathrm{site}\ \mathrm{methylation}\\ {}=\overline{\upbeta_0+{\upbeta}_1\ast \mathrm{age}+{\upbeta}_2\ast \mathrm{sex}+{\upbeta}_3\ast \mathrm{center}+{\upbeta}_4\ast \mathrm{smoking}}+\overline{\overline{\upbeta_5\ast \mathrm{family}}}\end{array}} $$

Subsequently, we used an elastic net approach to find the SNPs associated with the methylation marker with a coefficient that is statistically significantly different from zero. We set the elastic net algorithm to the following options: alpha = 0.5, lambda = lambda.min obtained from the cross-validation model and the seed = “123”.

*Elastic net model:* CpG site methylation^∗^ = β_0_ + β_1_ ∗ SNP_1_ + β_2_ ∗ SNP_2_… + β_*n*_ ∗ SNP_*n*_ where *CpG site methylation*^∗^ is the residual from the previous equation, and *n* refers to all the SNPs located ±50 kb from the CpG site that are not directly on the probe.

We tested the relationship between our selected *cis*-meQTL and the CpG site methylation as follows:

$$ {\displaystyle \begin{array}{l}\mathrm{CpG}\ \mathrm{site}\ \mathrm{methylation}\\ {}=\overline{\upbeta_0+{\upbeta}_1\ast \mathrm{meQTL}+{\upbeta}_2\ast \mathrm{age}+{\upbeta}_3\ast \mathrm{sex}+{\upbeta}_4\ast \mathrm{center}+{\upbeta}_5\ast \mathrm{smoking}}+\overline{\overline{\upbeta_6\ast \mathrm{family}}}\end{array}} $$

Once the SNPs were selected, we created and standardized a PRS-M using the approach outlined in our description of PRS-L above.

Subsequently, we applied the TSLS approach with lipids as the outcome to estimate the causal effect of DNA methylation on lipids, and tested whether PRS-M was related to lipids independently of predicted methylation.

As the final step, we tested for net pleiotropic effects using the MR-Egger test implemented in the *MendelianRandomization:R* package [16].

## Results

### Associations between DNA methylation and lipids

After removing the individuals with missing data, 993 individuals remained in the analyses. Of all tested CpG sites, five (cg00574958, cg11024682, cg07504977, cg06690548, and cg06500161) were associated with TGs (Table 1) and none were associated with HDL-C in GAW20 data (data not shown). Consequently, all subsequent analyses were restricted to the TG phenotype.

**Table 1:** Summary of the statistically significant results in the GAW20 data

TG–DNA methylation					
CpG	Chr: Pos	Gene	Β	SE	*p* Value
cg06690548	Chr4: 139162809	*SLC7A11*	−0.97	0.07	2.99 × 10^−4^
cg07504977	Chr10: 102131013	NA	0.36	0.008	6.19 × 10^−5^
cg00574958	Chr11: 68607622	*CPT1A*	−1.00	0.02	1.29 × 10^−14^
cg11024682	Chr17: 17730095	*SREBF1*	0.53	0.02	4.71 × 10^−5^
cg06500161	Chr21: 43656587	*ABCG1*	0.54	0.02	5.61 × 10^−4^
TG–PRS-L					
			Β	SE	*p* Value
PRS-L			0.05	7.68 × 10^− 6^	5.99 × 10^−9^
DNA methylation–Predicted TG					
CpG	Chr: Pos	Gene	Β	SE	*p* Value
cg06690548	Chr4: 139162809	*SLC7A11*	−0.05	2.86 × 10^−4^	2.84 × 10^− 3^
cg00574958	Chr11: 68607622	*CPT1A*	−0.12	1.17 × 10^− 3^	3.97 × 10^−4^
DNA methylation–PRS-M					
	CpG	Gene	Β	SE	*p* Value
PRS-M_CPT1A_	cg00574958	*CPT1A*	0.006	5.80 × 10^−6^	1.08 × 10^−2^
PRS-M_ABCG1_	cg06500161	*ABCG1*	0.008	3.77 × 10^−6^	2.61 × 10^−5^
TG–Predicted Methylation					
CpG	Chr: Pos	Gene	Β	SE	*p* Value
cg00574958	Chr11: 68607622	*CPT1A*	−2.36	0.37	1.28 × 10^−4^

### Causal effects of lipids on DNA methylation

Data from 655 individuals were available for MR analyses. The polygenic risk score for TG was robustly associated with the trait and associated with methylation of 2 (cg00574958 and cg06690548) of the 5 CpG sites (see Table 1). PRS-L was not associated with methylation of these 2 loci independently of the predicted TG levels (data not shown). PRS-L was not significantly associated with the other CpG sites. Thus, those results do not support a causal effect of TG on DNA methylation at cg07504977, cg11024682, and cg06500161.

### Causal effects of DNA methylation on lipids

We implemented the elastic net approach and created 2 PRS-Ms for cg00574958 (*CPT1A*; 3 SNPs), and cg06500161 (*ABCG1*; 5 SNPs) (see Table 1). The respective PRS-Ms were associated with the methylation of the cg00574958 and cg06500161 sites (see Table 1). The predicted methylation of the cg00574958 was associated with TG (see Table 1), but predicted methylation of the cg06500161 was not associated with TG (*p* value = 0.47).

### Pleiotropic effects

We tested the pleiotropy for the genetic instruments for the cg00574958 (*CPT1A*) using the MR-Egger test, which suggested no pleiotropic effect across the genetic variants in PRS-L and PRS-M for cg00574958.

## Discussion

Using GAW20 data, we assessed causal relations between fasting blood lipids and methylation from lipids to methylation. We observed causal effects of lipids on 2 methylation loci, but we could only investigate reverse causation for 1 locus because of the lack of appropriate instruments. The estimated associations between methylation and lipids were consistent with previous observational studies [4–6], but our conclusions diverged from prior MR findings [7].

Specifically, we established that methylation levels of cg00574958 (*CPT1A*) and cg06690548 (*SLC7A11*) can be affected by circulating TGs. The largest study of lipid epigenomics to date [7] also reported a causal effect of TGs on the methylation of the *CPT1A* locus, but not vice versa (ie, from cg00574958 methylation to TG). In contrast, we present novel evidence for a causal effect of cg00574958 methylation on fasting TGs. Our comprehensive bidirectional approach was enabled by a novel application of elastic net models to create a comprehensive polygenic methylation score. Although we were able to replicate and expand on the *CPT1A* finding, we did not detect other previously reported causal effects, possibly as a result of our smaller sample size: TG ➔ cg11024682 and TG/HDL ➔ cg06500161 [7]; additionally, we did not have robust genetic instruments to interrogate DNA methylation effects on lipids for other loci.

All 2 regions harboring CpG sites that emerged as causally associated in our analyses have extensive biological implications for lipid homeostasis. *CPT1A* encodes the liver isoform of carnitine palmitoyltransferase 1, a key enzyme in the fatty acid metabolism pathway; the cg00574958 locus specifically has been linked to plasma lipid levels [4–7] and lipoprotein subfractions [17]. In the same way, the *SLC7A11* (Solute carrier family 7 member 11) has been related to TGs [6, 7] and it has an important role protecting cells from oxidative stress [18].

## Conclusions

To conclude, we cannot rule out either direction of association between DNA methylation loci (namely in *CPT1A*) and TG blood levels, illustrating the complexity of biological regulation of lipid traits. Our findings likely paint only a part of the underlying causal picture. We did not have strong genetic instruments to test reverse causation for other lipid-associated CpG sites, highlighting the limitations of MR. Future studies should consider expanding the regions included in the elastic net (eg, to ±100 kb) and integrating publicly available bioinformatics data to improve the capture of *cis*-meQTLs to create robust genetic instruments for DNA methylation.
